# Relationship Between Hospital Volume and Outcomes of Minimally Invasive Esophagectomy for Esophageal Cancer: Analysis of the National Clinical Database in Japan

**DOI:** 10.1002/ags3.70229

**Published:** 2026-05-07

**Authors:** Soji Ozawa, Hiraku Kumamaru, Yuko Kitagawa, Kazuo Koyanagi, Junya Oguma, Harushi Udagawa, Hiroaki Miyata, Yasushi Toh, Hisahiro Matsubara

**Affiliations:** ^1^ Department of Surgery Tamakyuryo Hospital Machida Japan; ^2^ Tokai University School of Medicine Isehara Japan; ^3^ Department of Health Data Science, Yokohama City University Graduate School of Data Science Yokohama Japan; ^4^ Department of Surgery Keio University School of Medicine Tokyo Japan; ^5^ Department of Gastroenterological Surgery Tokai University School of Medicine Isehara Japan; ^6^ Department of Gastrointestinal and Pediatric Surgery Tokyo Medical University Tokyo Japan; ^7^ Department of Gastroenterological Surgery Toranomon Hospital Tokyo Japan; ^8^ Keio University School of Medicine Tokyo Japan; ^9^ Department of Gastrointestinal Surgery NHO Kyushu Cancer Center Fukuoka Japan; ^10^ Department of Frontier Surgery, Graduate School of Medicine Chiba University Chiba Japan

**Keywords:** complication, hospital volume, minimally invasive esophagectomy, mortality, outcome

## Abstract

**Aim:**

While esophagectomy is a high‐difficulty procedure, minimally invasive esophagectomy (MIE) requires even more advanced surgical skills, necessitating analysis of factors affecting surgical outcomes. This study examined the relationship between the postoperative mortality and complication rates of MIE and the annual hospital volume of esophagectomy to identify a threshold volume serving as a safety benchmark.

**Methods:**

We analyzed data from 22 712 patients registered in the National Clinical Database who underwent esophagectomy (MIE or open esophagectomy [OE]) at 1 037 hospitals in Japan between January 1, 2012, and December 31, 2015. Hospitals were stratified into five categories according to average annual volume of esophagectomies (MIE and OE). The primary outcome was operative mortality, and the secondary outcome was the incidence of postoperative complications.

**Results:**

The 22 712 cases consisted of 9 739 cases (42.9%) of MIE and 12 973 cases (57.1%) of OE. Regarding MIE outcomes, the overall crude perioperative mortality and perioperative complication rates were 2.1% and 40.9%, respectively. Multivariable analysis performed using very‐high‐volume hospitals as reference revealed an odds ratio for mortality in very‐low‐volume hospitals of 2.70 (*p* < 0.0001).

**Conclusion:**

The postoperative mortality rate in patients undergoing MIE was relatively low in hospitals with a higher annual average volume of esophagectomy cases. Mortality was significantly higher in hospitals performing five or fewer esophagectomies annually. However, residual confounding due to differences in baseline patient characteristics cannot be excluded, although key clinical factors were adjusted for. Therefore, careful attention must be paid to the surgical and perioperative management techniques when performing MIE, particularly in very‐low‐volume hospitals.

## Graphical abstract

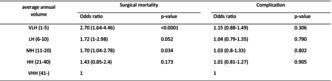

## 1 Introduction

Endoscopic surgery for esophageal cancer (minimally invasive esophagectomy; MIE) is aimed at minimizing damage to the body wall and reducing the surgical invasiveness. Since the first case report of MIE published by Cuschieri et al. in 1992, this approach for esophagectomy has spread rapidly worldwide [1]. According to a survey conducted by the Japan Society for Endoscopic Surgery, the first four cases of MIE for esophageal cancer in Japan were registered in 1993 [2]. According to the Annual Reports of the Japanese Association for Thoracic Surgery, the rates of MIE as assessed every four years were 2% in 1996, 6% in 2000, 11% in 2004, 21% in 2008, 34% in 2012, and 57% in 2016, indicating a steady and rapid increase in the adoption of MIE [3, 4, 5, 6, 7, 8]. Takeuchi et al. conducted a detailed analysis of the short‐term outcomes in 3 515 cases of MIE registered in the National Clinical Database (NCD) in 2011 and 2012. They reported a surgical mortality rate of MIE of 2.5% and complication rate of MIE of 41.9%, both of which were comparable to the corresponding rates reported for open esophagectomy (OE) [9].

Although esophagectomy is itself classified as a high‐difficulty procedure, MIE requires even more advanced surgical skills, thus necessitating an analysis of the factors affecting the postoperative outcomes of this procedure. The relationship between hospital surgical volume and surgical mortality was first reported by Luft et al. in 1979, covering 12 types of surgical procedures, including gastrointestinal surgeries, although esophagectomy was not among them [10]. Later, in 1998, Begg et al. examined this relationship specifically for esophagectomy, categorizing hospitals by the annual case volume as those performing 1–5 cases/year, 6–10 cases/year, and 11 or more cases/year [11]. Subsequently, Swisher et al. in 2000 [12], van Lanschot et al. in 2001 [13], and Birkmeyer et al. in 2002 [14] also reported on the relationship between the hospital case volume and mortality rate for esophagectomy, although each used a different case volume categorization.

In Japan, Kazui et al. reported on the relationship between the hospital case volume and mortality for esophagectomy in 2007 [15], based on pooled data from the annual reports of the Japanese Association for Thoracic Surgery between 2000 and 2004. The proportion of MIE cases during this period ranged from 6% to 11%, and the report included results for both OE and MIE [4, 5]. Nishigori et al. also analyzed the relationship between the hospital case volume and mortality in patients registered in the NCD who underwent esophagectomy with reconstruction between 2011 and 2013 [16].

Following previous studies, the present research was focused on the hospital case volume as a factor influencing the outcomes of MIE. While the relationship between hospital volume and mortality has been reported for cases of OE alone or mixed cases of OE plus MIE, this relationship remains unclear for cases of MIE alone. Therefore, this study was aimed at examining the relationship between the postoperative mortality and complication rates of MIE and the annual volume of esophagectomies performed at the hospital, to identify a threshold volume of annual cases that could serve as a safety benchmark.

## 2 Methods

The NCD is a nationwide registry in Japan, developed in cooperation with the surgical board certification system [17]. This web‐based data management system ensures data traceability by continuously involving individuals responsible for data verification, departmental reporting, and data entry. The NCD program defines postoperative outcomes rigorously, including morbidity and mortality. All variable definitions and inclusion criteria are publicly available via the NCD website (http://www.ncd.or.jp/) [17]. Clinical staging of esophageal cancer is performed preoperatively in accordance with the TNM classification system established by the Union for International Cancer Control (UICC) [18].

In this study, we included patients with thoracic esophageal cancer who underwent transthoracic subtotal esophagectomy with organ reconstruction. The exclusion criteria were as follows: Patients without esophageal cancer or with benign disease. MIE was defined as either a totally thoracoscopic and laparoscopic approach or a hybrid approach combining thoracoscopy with open laparotomy. Based on these criteria, a total of 22 712 cases who underwent esophagectomy at 1 037 hospitals between January 1, 2012, and December 31, 2015, were included in the present study.

Hospitals were stratified into five categories based on their average annual volume of esophagectomies (MIE plus OE), as follows: very‐low‐volume hospitals (VLH, 1–5 cases/year); low‐volume hospitals (LH, 6–10 cases/year); medium‐volume hospitals (MH, 11–20 cases/year); high‐volume hospitals (HH, 21–40 cases/year); and very‐high‐volume hospitals (VHH, ≥ 41 cases/year).

The primary outcome of this study was the operative mortality of MIE, stratified by the hospital volume. Operative mortality was defined as death occurring during the index hospitalization (regardless of the length of stay, up to 90 days), death occurring within 30 days after surgery, or death occurring within 30 days post‐discharge. The secondary outcome of the study was the incidence of postoperative complications following MIE. We also conducted exploratory analyses on the association between the volume categories vs. individual complication events using multivariable hierarchical regression with the same covariates as the mortality and complication analyses. Perioperative complications were defined as the occurrence of any of the following events: reoperation, any surgical site infection, wound dehiscence, anastomotic leakage, postoperative pneumonia, unplanned reintubation, pulmonary embolism, prolonged mechanical ventilation (≥ 48 h), renal dysfunction, urinary tract infection, peripheral nerve injury, cardiac arrest, myocardial infarction, postoperative blood transfusion, sepsis, atelectasis, empyema, chylothorax, recurrent laryngeal nerve paralysis, tracheal necrosis, or heart failure.

## 3 Statistical Analysis

We evaluated the number of surgeries performed at hospitals by volume category, stratified by surgical approach (MIE vs. OE). We further examined the characteristics of patients who underwent MIE procedures at these hospitals, as well as periprocedural factors such as operative time and blood loss. In addition, we assessed the crude incidence of the primary outcome and postoperative complications within each volume category. Hierarchical logistic regression models were constructed to evaluate the association between hospital volume category and the incidence of the primary outcome and operative complications among the MIE patients, adjusting for baseline patient characteristics. We did not perform statistical testing of baseline characteristics, in accordance with recommendations from the reporting guidelines such as STROBE [19]. All patient‐level factors of concern (e.g., age, ASA class, comorbidities, and preoperative treatments) were included as covariates in the hierarchical regression models. A *p*‐value of < 0.05 was considered statistically significant. All analyses were conducted using SAS version 9.4 (SAS Institute, Cary, NC, USA).

## 4 Results

Based on the average annual case volume, hospitals were classified into five categories: very‐low‐volume hospitals (VLH; *n* = 775; 74.7%); low‐volume hospitals (LH; *n* = 114; 11.0%); medium‐volume hospitals (MH; *n* = 81; 7.8%); high‐volume hospitals (HH; *n* = 41; 4.0%); and very‐high‐volume hospitals (VHH; *n* = 26; 2.5%) (Table 1). Of the total of 22 712 cases included in this study, 9 739 cases (42.9%) had undergone MIE, and 12 973 cases (57.1%) had undergone OE. The proportion of MIE cases in each hospital volume category was as follows: VLH, 27.9%; LH, 40.7%; MH, 49.7%; HH, 48.3%; and VHH, 45.0% (Table 1). The patient background characteristics of the MIE cohort are shown in Table 2.

**TABLE 1:** Hospital volume and type of surgery.

Average annual volume												
	VLH (1–5)		LH (6–10)		MH (11–20)		HH (21–40)		VHH (41–)		Total	
Hospital	775	(74.7%)	114	(11.0%)	81	(7.8%)	41	(4.0%)	26	(2.5%)	1 037	(100.0%)
MIE	1131	(27.9%)	1168	(40.7%)	2055	(49.7%)	2074	(48.3%)	3311	(45.0%)	9739	(42.9%)
OE	2926	(72.1%)	1701	(59.3%)	2076	(50.3%)	2220	(51.7%)	4050	(55.0%)	12973	(57.1%)
Total	4057	100.0%	2869	100.0%	4131 1	100.0%	4294	100.0%	7361	100.0%	22 712	(100.0%)

Abbreviations: HH, high‐volume hospitals; LH, low‐volume hospitals; MH, medium‐volume hospitals; VHH, very‐high‐volume hospitals; VLH, very‐low‐volume hospitals.

**TABLE 2:** Patient background.

Average annual volume												
	VLH (1–5)		LH (6–10)		MH (11–20)		HH (21–40)		VHH (41–)		Total	
*N*	1 131		1 168		2 055		2 074		3 311		9 739	
Age												
< 60	181	(16.0%)	208	(17.8%)	379	(18.4%)	403	(19.4%)	709	(21.4%)	1 880	(19.3%)
60–69	458	(40.5%)	494	(42.3%)	854	(41.6%)	887	(42.8%)	1 366	(41.3%)	4 059	(41.7%)
70–79	438	(38.7%)	407	(34.8%)	707	(34.4%)	695	(33.5%)	1 118	(33.8%)	3 365	(34.6%)
80 and above	54	(4.8%)	59	(5.1%)	115	(5.6%)	89	(4.3%)	118	(3.6%)	435	(4.5%)
Female	185	(16.4%)	175	(15.0%)	339	(16.5%)	343	(16.5%)	522	(15.8%)	1 564	(16.1%)
Diabetes	166	(14.7%)	136	(11.6%)	272	(13.2%)	281	(13.5%)	388	(11.7%)	1 243	(12.8%)
Smoking	454	(40.1%)	445	(38.1%)	805	(39.2%)	830	(40.0%)	1 301	(39.3%)	3 835	(39.4%)
Habitual alcohol	634	(56.1%)	736	(63.0%)	1 252	(60.9%)	1 396	(67.3%)	2 254	(68.1%)	6 272	(64.4%)
Dyspnea	15	(1.3%)	24	(2.1%)	30	(1.5%)	24	(1.2%)	21	(0.6%)	114	(1.2%)
ADL support	20	(1.8%)	22	(1.9%)	44	(2.1%)	37	(1.8%)	17	(0.5%)	140	(1.4%)
COPD	80	(7.1%)	88	(7.5%)	138	(6.7%)	124	(6.0%)	330	(10.0%)	760	(7.8%)
Hypertension	443	(39.2%)	425	(36.4%)	751	(36.5%)	718	(34.6%)	1 031	(31.1%)	3 368	(34.6%)
Dialysis	3	(0.3%)	1	(0.1%)	5	(0.2%)	3	(0.1%)	5	(0.2%)	17	(0.2%)
Weight loss	102	(9.0%)	87	(7.4%)	148	(7.2%)	129	(6.2%)	133	(4.0%)	599	(6.2%)
Preoperadiation	29	(2.6%)	38	(3.3%)	96	(4.7%)	112	(5.4%)	185	(5.6%)	460	(4.7%)
Preopechemotherapy	273	(24.1%)	221	(18.9%)	366	(17.8%)	411	(19.8%)	617	(18.6%)	1 888	(19.4%)
ASA3 and above	103	(9.1%)	93	(8.0%)	132	(6.4%)	105	(5.1%)	156	(4.7%)	589	(6.0%)
Past PCI	25	(2.2%)	17	(1.5%)	40	(1.9%)	25	(1.2%)	57	(1.7%)	164	(1.7%)
Surgical T												
T0	9	(0.8%)	9	(0.8%)	11	(0.5%)	36	(1.7%)	83	(2.5%)	148	(1.5%)
Tis	447	(39.5%)	509	(43.6%)	845	(41.1%)	923	(44.5%)	1 558	(47.1%)	4 282	(44.0%)
T1	178	(15.7%)	165	(14.1%)	283	(13.8%)	309	(14.9%)	498	(15.0%)	1 433	(14.7%)
T2	425	(37.6%)	416	(35.6%)	773	(37.6%)	694	(33.5%)	1 035	(31.3%)	3 343	(34.3%)
T3	48	(4.2%)	49	(4.2%)	125	(6.1%)	95	(4.6%)	107	(3.2%)	424	(4.4%)
T4	12	(1.1%)	8	(0.7%)	9	(0.4%)	6	(0.3%)	15	(0.5%)	50	(0.5%)
TX	12	(1.1%)	12	(1.0%)	9	(0.4%)	11	(0.5%)	15	(0.5%)	59	(0.6%)
Surgical *N*												
N0	573	(50.7%)	586	(50.2%)	962	(46.8%)	1051	(50.7%)	1 727	(52.2%)	4 899	(50.3%)
N1	275	(24.3%)	284	(24.3%)	492	(23.9%)	558	(26.9%)	948	(28.6%)	2 557	(26.3%)
N2	198	(17.5%)	202	(17.3%)	417	(20.3%)	331	(16.0%)	454	(13.7%)	1 602	(16.4%)
N3	74	(6.5%)	86	(7.4%)	169	(8.2%)	120	(5.8%)	168	(5.1%)	617	(6.3%)
NX	11	(1.0%)	10	(0.9%)	15	(0.7%)	14	(0.7%)	14	(0.4%)	64	(0.7%)
Metastasis	23	(2.0%)	20	(1.7%)	43	(2.1%)	34	(1.6%)	69	(2.1%)	189	(1.9%)

*Note:* Weight loss was defined as a reduction of ≥ 10% in body weight within the previous 6 months. Smoking status and other factors, including habitual alcohol, were classified as present or absent just prior to the surgery.

Abbreviations: HH, high‐volume hospitals; LH, low‐volume hospitals; MH, medium‐volume hospitals; VHH, very‐high‐volume hospitals; VLH, very‐low‐volume hospitals.

The outcomes of MIE were as follows: the overall crude perioperative mortality rate was 2.1%. The mortality rates by the hospital volume category were: VLH, 3.9%; LH, 2.5%; MH, 2.5%; HH, 1.9%; and VHH, 1.2%. The overall crude perioperative complication rate was 40.9%, and the rates by hospital volume category were as follows: VLH, 44.2%; LH, 40.8%; MH, 41.0%; HH, 42.5%; and VHH, 38.7% (Table 3). In terms of perioperative outcomes, the incidence of postoperative pneumonia ranged from 12.2% to 15.7% across groups, atelectasis from 3.2% to 5.0%, recurrent laryngeal nerve paralysis from 9.7% to 13.6%, chylothorax from 2.0% to 3.0%, and anastomotic leakage from 10.7% to 18.0% (Table 4).

**TABLE 3:** Surgical mortality and complications.

Average annual volume												
	VLH (1–5)		LH (6–10)		MH (11–20)		HH (21–40)		VHH (41–)		Total	
*N*	1 131		1 168		2 055		2 074		3 311		9 739	
Surgical mortality												
(+)	44	(3.9%)	29	(2.5%)	52	(2.5%)	39	(1.9%)	40	(1.2%)	204	(2.1%)
(−)	1 087	(96.1%)	1 139	(97.5%)	2 003	(97.5%)	2 035	(98.1%)	3 271	(98.8%)	9 535	(97.9%)
Complication												
(+)	500	(44.2%)	477	(40.8%)	843	(41.0%)	882	(42.5%)	1 282	(38.7%)	3 984	(40.9%)
(−)	631	(55.8%)	691	(59.2%)	1 212	(59.0%)	1 192	(57.5%)	2 029	(61.3%)	5 755	(59.1%)

Abbreviations: HH, high‐volume hospitals; LH, low‐volume hospitals; MH, medium‐volume hospitals; VHH, very‐high‐volume hospitals; VLH, very‐low‐volume hospitals.

**TABLE 4:** Perioperative outcomes.

Average annual volume					
	VLH (1–5)	LH (6–10)	MH (11–20)	HH (21–40)	VHH (40–)
*N*	1 131	1 168	2 055	2 074	3 311
Opetime (min) (median (IQR))	543 (338–799)	560 (350–812)	553 (345–829)	535 (355–759)	486 (259–749)
Bloodlost (ml) (median (IQR))	250 (20–1 300)	238 (30–1 135)	228.5 (22–1 085)	230 (30–925)	260 (42–1 100)
Resection margin					
R0	1 051 (92.9%)	1 107 (94.8%)	1 909 (92.9%)	1 966 (94.8%)	3 195 (96.5%)
R1	40 (3.5%)	31 (2.7%)	74 (3.6%)	54 (2.6%)	58 (1.8%)
R2	31 (2.7%)	18 (1.5%)	61 (3.0%)	43 (2.1%)	49 (1.5%)
Rx	9 (0.8%)	12 (1.0%)	11 (0.5%)	11 (0.5%)	9 (0.3%)
Postoperative pneumonia	177 (15.7%)	143 (12.2%)	269 (13.1%)	280 (13.5%)	426 (12.9%)
Atelectasis	57 (5.0%)	37 (3.2%)	80 (3.9%)	79 (3.8%)	110 (3.3%)
RLNP	110 (9.7%)	123 (10.5%)	251 (12.2%)	282 (13.6%)	397 (12.0%)
Chylothorax	30 (2.7%)	35 (3.0%)	41 (2.0%)	49 (2.4%)	54 (2.0%)
Anastomotic leakage	204 (18.0%)	166 (14.2%)	253 (12.3%)	288 (13.9%)	354 (10.7%)
PLOS (days) (median (IQR))	30 (19–52)	27 (19–47)	26 (18–45)	24 (17–38)	21 (16–32)

Abbreviations: PLOS, post‐operative length of stay; RLNP, recurrent laryngeal nerve paralysis.

According to multivariable analysis conducted using a hierarchical logistic regression model, using VHH as the reference group, the odds ratios for mortality by hospital volume category were as follows: VLH, 2.70 (*p* < 0.0001); LH, 1.72 (*p* = 0.052); MH, 1.70 (*p* = 0.034); and HH, 1.43 (*p* = 0.173). The odds ratios for surgical complications were as follows: VLH, 1.15 (*p* = 0.306); LH, 1.04 (*p* = 0.790); MH, 1.03 (*p* = 0.802); and HH, 1.01 (*p* = 0.905) (Table 5).

**TABLE 5:** Multivariable analysis for surgical mortality and complications.

Average annual volume	Surgical mortality		Complication	
	Odds ratio	*p*	Odds ratio	*p*
VLH (1–5)	2.70 (1.64–4.46)	< 0.0001	1.15 (0.88–1.49)	0.306
LH (6–10)	1.72 (1–2.98)	0.052	1.04 (0.79–1.35)	0.790
MH (11–20)	1.70 (1.04–2.78)	0.034	1.03 (0.8–1.33)	0.802
HH (21–40)	1.43 (0.85–2.4)	0.173	1.01 (0.81–1.27)	0.905
VHH (41–)	1		1	

Abbreviations: HH, high‐volume hospitals; LH, low‐volume hospitals; MH, medium‐volume hospitals; VHH, very‐high‐volume hospitals; VLH, very‐low‐volume hospitals.

The association between hospital volume and the incidence of five complications—four thoracic procedure–related complications and anastomotic leakage (one of the three major complications)—was examined using hierarchical logistic regression models (Table 6). In the multivariable analysis using VHH as the reference, hospital volume was not significantly associated with postoperative pneumonia, recurrent laryngeal nerve paralysis, or chylothorax. VLH were associated with a significantly increased risk of atelectasis (odds ratio [OR], 2.03; 95% confidence interval [CI], 1.05–3.94). In contrast, the risk of anastomotic leakage was significantly higher in VLH (OR, 2.23; 95% CI, 1.51–3.30), LH (OR, 1.66; 95% CI, 1.15–2.41), and HH (OR, 1.41; 95% CI, 1.05–1.90) compared with VHH, whereas no significant association was observed in MH.

**TABLE 6:** Multivariable analysis for complications.

Average annual volume	Postoperative pneumonia	Atelectasis	RLNP	Chylothorax	Anastomotic leakage
	Odds ratio	Odds ratio	Odds ratio	Odds ratio	Odds ratio
VLH (1–5)	1.34 (0.92–1.97)	2.03 (1.05–3.94)	0.82 (0.50–1.35)	1.18 (0.62–2.24)	2.23 (1.51–3.30)
LH (6–10)	1.02 (0.71–1.47)	1.35 (0.71–2.56)	0.90 (0.56–1.44)	1.42 (0.79–2.53)	1.66 (1.15–2.41)
MH (11–20)	0.96 (0.70–1.30)	1.31 (0.77–2.24)	1.17 (0.79–1.74)	0.94 (0.57–1.54)	1.34 (0.97–1.86)
HH (21–40)	0.93 (0.70–1.24)	1.05 (0.63–1.73)	0.88 (0.62–1.25)	1.14 (0.72–1.79)	1.41 (1.05–1.90)
VHH (41–)	1	1	1	1	1

Abbreviations: HH, high‐volume hospitals; LH, low‐volume hospitals; MH, medium‐volume hospitals; RLNP, recurrent laryngeal nerve paralysis; VHH, very‐high‐volume hospitals; VLH, very‐low‐volume hospitals.

## 5 Discussion

The existence of a relationship between the hospital surgical volume and surgical mortality was first reported by Luft et al. in 1979, although their analysis included several surgeries other than esophagectomy [10]. For the case of esophagectomy, Begg et al. categorized hospitals according to the surgical case volume into three groups—hospitals performing 1–5 cases/year, 6–10 cases/year, and ≥ 11 cases/year—and reported the existence of an association between the case volume and the mortality [11]. Other previous studies have used varying categorizations of the hospital case volume: Swisher et al. classified hospitals into two groups (hospitals performing 1–4 cases/year or ≥ 5 cases/year) [12], van Lanschot et al. classified them into three groups (hospitals performing 1–10 cases, 11–20 cases or ≥ 21 cases/year) [13], and Birkmeyer et al. used five categories (hospitals performing 0–1, 2–4, 5–7, 8–19, or ≥ 20 cases/year) [14]. Despite the different categorizations, the lowest volume hospitals consistently showed higher mortality rates. Consistent with these previous reports, in our study, multivariable analysis revealed a significantly higher mortality risk in the VLH group. Previous studies, including those from Japan, have focused on either cases of OE alone or combined cases of OE plus MIE. We limited our analysis to cases of MIE alone, and our results demonstrated that the mortality remained higher in very‐low‐volume hospitals even for MIE procedures.

The similarity in the relationship of the hospital case volume with mortality between MIE and OE is likely attributable to two main factors: first, the core surgical techniques, such as esophageal mobilization and lymph node dissection within the mediastinum, are fundamentally the same; and second, the principles of perioperative management remain essentially consistent between the two approaches.

There are differing viewpoints on how to define low‐volume hospitals that are associated with higher mortality rates. The Leapfrog Group in the U.S. recommended a minimum volume standard of 7 cases per year in 2000 [20], 13 in 2003 [20], and 20 in 2018 [21]. The thresholds proposed by this group may be overly restrictive and may not consider real‐world factors such as individual patient characteristics and surgical risk at the time of esophagectomy [21]. In Japan, Fujita et al. analyzed 31 380 cases of esophagectomy performed between 2001 and 2006, categorizing hospitals according to the case volume into six groups: hospitals performing ≤ 4, 5–9, 10–19, 20–39, 40–79, and ≥ 80 cases/year. They identified hospitals performing fewer than 5 cases per year as low‐volume hospitals associated with higher mortality rates [22]. In the present study, we also defined hospitals performing ≤ 5 MIE cases per year as low‐volume hospitals, which aligns closely with the previous classification. Such hospitals perform surgery only once every 2–3 months on average, indicating infrequent experience.

In addition to the hospital volume, the surgeon volume (annual number of surgeries performed by the surgeon) has also been shown as an important predictor of mortality. In 2003, Birkmeyer et al. reported an inverse correlation between the surgeon volume and surgical mortality [23]. The adjusted odds ratio for mortality associated with esophagectomy was 2.30 for low‐volume surgeons as compared with high‐volume surgeons. Surgeon volume appears to explain a large proportion (46%) of the effect of hospital volume on the surgical outcomes. The mortality was higher in patients operated upon by low‐volume surgeons, regardless of the hospital's surgical volume. In Japan, Nishigori et al. found a relationship between the hospital case volume and outcomes, but not between the surgeon volume and outcomes [16]. This has been attributed to factors such as multidisciplinary meetings held at high‐volume hospitals and the involvement of experienced senior surgeons who supervise and support less experienced colleagues, both for surgical technique and postoperative care.

In addition to short‐term outcomes such as mortality, long‐term outcomes have also been investigated. Brusselaers et al. conducted a meta‐analysis of long‐term survival after esophagectomy and found that the survival rates were 18%–25% higher in high‐volume hospitals and 9%–13% for high‐volume surgeons, as compared with their low‐volume counterparts [24]. Even after exclusion of early deaths, a 15% survival benefit was observed, indicating that the difference in survival was not solely due to lower early postoperative mortality. They noted that the surgeon volume had a stronger impact on the long‐term survival than the hospital case volume.

In this study, we identified a significant association between the hospital case volume and the surgical mortality in patients undergoing MIE, with the mortality rates being significantly higher in hospitals performing ≤ 5 MIEs annually. However, no such association was found for the surgical complication rates. Abdelsattar et al. defined failure to rescue (FTR) as death following a major postoperative complication, and reported that at the hospital level, there were substantial differences in the FTR rates across hospital case volume quintiles. Specifically, 21.2% of patients who experienced complications died before discharge in low‐volume hospitals as compared with 13.4% in high‐volume hospitals [25]. This finding underscores the need for strategies to improve the recognition and management of complications, especially in high‐risk patients. The differences in FTR rates between low‐ and high‐volume hospitals could be attributed to two main factors available at high‐volume hospitals (1): evidence‐based processes of care aimed at timely and effective management of postoperative complications, and (2) hospital structural resources, such as the presence of dedicated surgical intensive care unit intensivists and multidisciplinary teams. In our study, although the complication rates did not differ significantly by the hospital case volume, the higher FTR rate in low‐volume hospitals reported in previous studies likely contributed to the increased mortality observed in these hospitals. An additional analysis of selected major complications (Table 6) indicated that atelectasis and anastomotic leakage were more frequent in very‐low‐volume hospitals, which may partly contribute to the observed volume–mortality relationship. The shorter postoperative length of stay (PLOS) observed in VHH (21 days) compared with VLH (30 days) may partly reflect more effective perioperative management, which could contribute to a lower FTR rate. There seems to be no apparent association between hospital volume and operative time or blood loss. Notably, no apparent association was observed between hospital volume and operative time or intraoperative blood loss. This finding may suggest that the technical aspects of MIE have been relatively standardized across institutions in Japan, whereas differences in postoperative management capacity and institutional resources may play a more critical role in determining surgical mortality.

According to the Annual Reports of the Japanese Association for Thoracic Surgery, the proportion of MIE cases increased from 2% in 1996 to 57% in 2016. Calculated at 4‐year intervals, the rates were 4%, 15%, 10%, 13%, and 23%, respectively, indicating that the study period (2012–2015) corresponded to a phase of rapid MIE adoption [3, 4, 5, 6, 7, 8]. Several factors could be considered as having contributed to this surge: MIE began to be covered by Japanese National Health Insurance in April 2002; adoption of the prone patient position for the thoracic phase of surgery increased from 11% in 2007 to 75% in 2017, contributing to improved technical ease of the procedure, particularly by facilitating better visualization of the surgical field; and technical proficiency in the surgical techniques was facilitated by frequent seminars [26]. Furthermore, in Japan, while the introduction of robot‐assisted MIE is subject to strict institutional and surgeon requirements, the adoption of MIE faced fewer regulatory hurdles prior to the establishment of the government's framework for highly advanced medical technologies in 2016, which also appears to have contributed to its rapid change. Thus, the study period from 2012 to 2015 was selected because it corresponds to a nationwide transitional phase in Japan during which MIE was rapidly adopted, while institutional experience and perioperative management systems varied substantially among hospitals.

Hospital volume was defined based on the total number of esophagectomies, including both minimally invasive and open procedures, to reflect overall institutional experience with complex esophagectomy and perioperative management. Although outcome analyses were limited to MIE cases, this approach was intended to capture hospital‐level expertise and structural capacity rather than procedure‐specific technical volume alone.

Notably, although the excess mortality risk was most pronounced in VLH, the adjusted odds ratios in LH and HH did not reach statistical significance, and the magnitude of risk observed in MH was substantially smaller than that in VLH. Furthermore, the overall incidence of postoperative complications was comparable across LH, MH, HH, and VHH groups. These findings suggest that the relationship between hospital volume and mortality is not strictly dichotomous and that outcomes in hospitals performing MIE on a regular basis may be broadly similar, except for very‐low‐volume institutions.

To reduce mortality, centralization of MIE to high‐volume centers has been proposed. However, this strategy could pose geographic limitations for patients, depending on the region they reside in. Therefore, uniformly setting a strict volume threshold may not be practically feasible. As an alternative, it is essential to also consider the surgeon volume and to ensure that the operation, as well as perioperative care, is undertaken by experts such as Board‐Certified Esophageal Surgeons by the Japan Esophageal Society.

This study had several limitations. First, although the analysis focused on the hospital case volume, the quality of individual surgeons who performed the procedures at each institution remains unknown. Presence of/Supervision by a Board‐Certified Esophageal Surgeon by the Japan Esophageal Society, who is a specialist highly experienced in esophageal surgery, should also be considered. In fact, the case volume threshold for categorizing a hospital as low volume may be lower if such a certified surgeon is affiliated with the institution. Second, important institutional factors such as hospital size, availability of dedicated surgical intensive care units, intensivist involvement, and multidisciplinary perioperative management systems were not available in the NCD and could not be evaluated. These unmeasured factors may partially explain the observed volume–outcome relationship. Third, this study examined operative mortality as a short‐term outcome; however, it would have been more informative to evaluate the association between hospital case volume and the long‐term outcomes, which is the ultimate goal in the treatment of esophageal cancer. Finally, although we adjusted for major patient‐level characteristics, including age, ASA status, comorbidities, preoperative treatments, and tumor stage, residual confounding cannot be excluded. In particular, unmeasured factors such as patient frailty, nutritional status, and other aspects of physiological reserve may have influenced the observed outcomes.

In conclusion, the mortality rates of MIE were lower in hospitals with a higher annual average volume of esophagectomies; the overall incidence of perioperative complications showed a relatively weaker correlation with the hospital case volume. While failure to rescue may have contributed to the observed differences, the surgical mortality was significantly higher in hospitals that performed five or fewer esophagectomies annually (VLH). Therefore, careful attention must be paid to the surgical and perioperative management techniques when performing MIE, particularly in very‐low‐volume hospitals.

## Author Contributions

**Soji Ozawa:** conceptualization, methodology, writing – original draft, writing – review and editing. **Hiraku Kumamaru:** conceptualization, methodology, investigation, formal analysis, writing – review and editing. **Yuko Kitagawa:** conceptualization, methodology, writing – review and editing. **Kazuo Koyanagi:** conceptualization, methodology, writing – review and editing. **Junya Oguma:** conceptualization, methodology, writing – review and editing. **Harushi Udagawa:** conceptualization, methodology, writing – review and editing. **Hiroaki Miyata:** conceptualization, methodology, writing – review and editing. **Yasushi Toh:** conceptualization, methodology, writing – review and editing. **Hisahiro Matsubara:** conceptualization, methodology, writing – review and editing.

## Funding

The authors have nothing to report.

## Ethics Statement

Approval of the research protocol: This study was conducted in accordance with the Helsinki Declaration of 1964 and its later versions. This study was approved by the institutional review boards of Tamakyuryo Hospital (approval number: 25–1). Informed Consent: The requirement for informed consent was waived because all data included in the NCD were deidentified and anonymized.

## Conflicts of Interest

H.K. reports receiving a consultation fee from EPS Corporation, a speaker fee from Novartis Pharma K.K., and a research grant on an unrelated subject from Amgen K.K. and Pfizer K.K. H.K. is affiliated with the Department of Healthcare Quality Assessment at the University of Tokyo, a social collaboration department supported by the National Clinical Database, Johnson & Johnson K.K., Nipro Corporation, and Intuitive Surgical Sàrl. H.K. also receives research funding from the National Clinical Database for participating in clinical research. Y.K. is the Editor‐in‐Chief of the Annals of Gastroenterological Surgery. H.Ma. is a member of the editorial board of the Annals of Gastroenterological Surgery. Other authors declare no conflicts of interest related to this article.

## Data Availability

Research data are not shared.
